# Barriers and Facilitators to Endovascular Aortic Repair Follow-Up

**DOI:** 10.1001/jamanetworkopen.2025.46327

**Published:** 2025-12-02

**Authors:** Marissa C. Jarosinski, Lindsey A. Olivere, Jackie L. Barnes, Edith Tzeng, Nathan L. Liang, Kimberly J. Rak, Amanda R. Phillips

**Affiliations:** 1 Division of Vascular Surgery, University of Pittsburgh Medical Center, Pittsburgh, Pennsylvania; 2 Department of Critical Care Medicine, Clinical Research, Investigation, and Systems Modeling of Acute Illness Center, University of Pittsburgh School of Medicine, Pittsburgh, Pennsylvania; 3 Veterans Affairs Pittsburgh Health Services, Pittsburgh, Pennsylvania; 4 Oregon Health and Vascular Institute, Eugene

## Abstract

**Importance:**

The rate of follow-up after endovascular aortic repair (EVAR) of an abdominal aortic aneurysm (AAA) is low despite guidelines recommending regular surveillance due to increased risk of stent-related complications, rupture, and death.

**Objective:**

To identify existing barriers and facilitators to patient follow-up adherence after EVAR to inform future interventions to improve follow-up adherence.

**Design, Setting, and Participants:**

This qualitative study used purposive diversity sampling in a multisite, academic health system to interview patients who underwent elective or emergency EVAR between October 2019 and October 2021 and had varying follow-up patterns; health care professionals (HCPs), including vascular surgeons and advanced practice providers ([APPs] nurse practitioners and physician associates); and key clinical personnel, including administrative assistants, schedulers, nurses, and medical assistants with diverse backgrounds and experience with EVAR. Data were collected between December 2021 and February 2023. Data were analyzed between December 2022 and April 2025.

**Exposure:**

Participants engaged in a single semistructured phone interview. Interview guides and thematic analysis were informed by the Theoretical Domains Framework.

**Main Outcomes and Measures:**

Barriers and facilitators to patient follow-up adherence after EVAR.

**Results:**

The sample included 55 participants (25 patients [17 (68%) males], 16 HCPs [12 physicians and 4 APPs], and 14 key clinical personnel). Of the patients, 13 had complete follow-up and a median (IQR) age of 71.6 (63.8-75.6) years, and 12 had incomplete follow-up and a median (IQR) age of 72.0 (66.3-79.3) years. The HCPs had a median (range) 7 (3-16) years in practice, and key clinical personnel worked in their current position a median (range) 2 (1-4) years. Four main themes were identified during the qualitative analysis. In theme 1, patients’ perceptions of follow-up importance were primarily affected by beliefs about immediate consequences and physical reinforcements (ie, symptoms). In theme 2, despite attempts by HCPs to provide education, patients’ knowledge of anatomical concepts did not translate to better follow-up; however, explicit knowledge of clinical consequences (ie, rupture and death) did. In theme 3, patient memory and attention issues posed barriers to follow-up, but these barriers were partially mitigated by protocolization of follow-up processes (eg, appointment reminders, standardization of preoperative and postoperative resources, and optimization of discharge paperwork). In theme 4, social support and environmental circumstances, particularly transportation, were both substantial facilitators and barriers to follow-up.

**Conclusions and Relevance:**

In this qualitative study, patient focus on immediate outcomes after EVAR and memory and attention factors were important barriers to follow-up. However, recognition of explicit long-term consequences, presence of social support, and protocolization facilitated follow-up. Potential interventions to improve follow-up could include enhanced protocolization, a robust recall system to identify and contact patients with missed appointments, careful expansion of telemedicine, and transportation adjuncts.

## Key Points

**Question:**

What are existing barriers and facilitators to follow-up after endovascular aortic repair (EVAR) of abdominal aortic aneurysms (AAA)?

**Findings:**

This qualitative study used semistructured interviews of 55 health care professionals, key clinical personnel, and patients and found that patient perceptions of EVAR and memory and attention issues were barriers to follow-up, while existing social support and protocolization facilitated follow-up adherence.

**Meaning:**

This study suggests that targeted patient education, refined protocolization, and resources to target social disadvantage are needed to improve postoperative follow-up after EVAR.

## Teaser

This qualitative study assesses the barriers and facilitators to follow-up after endovascular aortic repair of abdominal aortic aneurysms.

## Introduction

Endovascular aortic repair (EVAR) is first-line therapy for abdominal aortic aneurysm (AAA) management due to reductions in short-term morbidity and mortality compared with open surgery.^1,2,3^ However, regular follow-up surveillance, postoperatively and annually thereafter, is recommended due to high rates of graft-related complications. Indeed, endoleak has been reported in up to 30% of patients and carries the potential for long-term sac reexpansion, rupture, and death.^2,4,5^

Up to 60% of patients are lost to follow-up after EVAR.^6^ This has been associated with increased mortality compared with patients who follow-up even once postoperatively.^5,6^ Factors associated with loss to follow-up include male sex, advanced age, lack of a primary care physician (PCP), long driving distance to the hospital, and nursing home discharge.^6,7,8,9,10^ Many of these factors are not modifiable, limiting initiatives to improve follow-up. Retrospective studies using clinical or administrative data also may fail to capture perceived barriers and facilitators to follow-up from the perspectives of patients, clinical staff, and surgeons.

The Theoretical Domains Framework (TDF) was developed to identify modifiable health professional behaviors and aid in the implementation of evidence-based recommendations.^11^ It primarily uses qualitative methods of data collection focused on 14 domains of influence associated with behavior change.^11,12^ The TDF has been extensively validated and cited in more than 800 publications in various health care settings.^11,13,14^ The objective of this study was to qualitatively analyze barriers and facilitators to follow-up after EVAR using the TDF to inform future implementation of interventions to improve patient follow-up adherence.

## Methods

The University of Pittsburgh’s Human Research Protection Office approved this qualitative study. Informed consent was obtained verbally before each interview. Study design and data collection followed the Consolidated Criteria for Reporting Qualitative Research (COREQ) guideline.^15^

### Recruitment

The study protocol with methods and rationale has been previously published.^16^ Purposive diversity sampling was used to recruit patients who underwent elective or emergency EVAR between October 2019 and October 2021; health care professionals (HCPs; eg, vascular surgeons) and advanced practice professionals (APPs; eg, nurse practitioners and physician associates); and key clinical personnel, including administrative assistants, schedulers, nurses, and medical assistants within the division of vascular surgery.^17^ Patients were stratified into complete and incomplete follow-up categories, with incomplete follow-up defined as missing at least 1 follow-up appointment.^16^ Eligible participants were recruited by telephone and/or email and offered $25 for participation.

### Data Collection

Patients’ demographic data were obtained from electronic health records (EHRs) and included age, sex, race, ethnicity, employment, educational level, and marital status. Demographic data for HCPs and key clinical personnel included sex, race, ethnicity, profession, years since training was completed, years in the current position, years at the current hospital, and the number of EVARs performed or patients interacted with annually. These data were collected during a telephone survey conducted immediately after semistructured interviews. Race and ethnicity were collected to assess the diversity of the included patients, HCPs, and key clinical personnel in line with purposive diversity sampling techniques.^17^ Patient race categories were predefined by the institutional electronic health record. Race categories, defined according to National Institutes of Health reporting standards, were offered to HCPs and key clinical personnel for self-selection.^18^

The study used semistructured interview guides based on the TDF that were previously developed and piloted by the study team.^16^ Telephone interviews were conducted between December 2021 and February 2023 by qualitative researchers with extensive health care interviewing experience (J.L.B. and K.J.R.) until thematic saturation was reached.^19^ Interviews were recorded, transcribed, and deidentified.

### Data Analysis

Codebook development was performed by the qualitative team (J.L.B., K.J.R., A.R.P., and L.A.O) using a hybrid inductive-deductive approach, examining data based on prespecified codes from the TDF, but also adding emergent codes (eTable 1 in Supplement 1). Two coders (L.A.O. and J.L.B.) applied codes to transcripts until inter-rater reliability was reached (≥0.7 Cohen κ). Disagreements were reconciled through consensus. After reaching sufficient agreement, the remaining data were then divided, and 2 team members independently coded the remaining transcripts (L.A.O. and J.L.B.). Inter-rater reliability was then checked at predetermined points based on the number of transcripts per participant group. Patient transcripts were checked after each quarter, key clinical personnel transcripts were checked halfway and at the end of group coding (twice), and HCP transcripts were checked after each third of the transcript coding was complete. All transcripts were coded or recoded using the final codebook. Thematic analysis was subsequently performed by the qualitative team (M.C.J., L.A.O., J.L.B., K.J.R., and A.R.P.), focusing on 5 TDF domains: knowledge; memory, attention, and decision processes; beliefs about consequences; environmental context and resources; and reinforcements. Codebook development and data analysis were conducted between December 2022 and April 2025. NVivo, version 14 (Lumivero) was used for data management and analysis.

## Results

Fifty-five participants were interviewed. Of 19 HCPs contacted, 16 agreed to participate (12 physicians, 4 APPs; 8 males [50%] and 8 females [50%]). All of these participants identified as non-Hispanic, 1 (6%) identified as Asian, 1 (6%) as Black, 13 (81%) as White, and 1 (6%) as multiracial. They had a median (range) of 7 (3-16) years in practice, and 8 physicians (50%) performed 5 to 20 EVARs per year (Table 1). Of 26 key clinical personnel contacted, 14 agreed to participate (3 nurses, 4 schedulers, 5 administrators, and 2 medical assistants; 12 females [86%] and 2 males [14%]). Of these individuals, 13 (93%) were non-Hispanic individuals and 1 was a Hispanic individual (7%), 1 (7%) identified as Black, 12 (86%) as White, and 1 (7%) declined to answer. The median (range) time worked in their current position was 2 (1-4) years, and 8 (57%) interacted with greater than 20 patients who underwent EVAR per year (Table 1).

**Table 1.** Demographics of Health Care Professionals and Key Clinical Personnel

Characteristic^a^	No. (%)^b^	
	HCP (n = 16)	Key clinical personnel (n = 14)
Sex		
Female	8 (50)	12 (86)
Male	8 (50)	2 (14)
Race^c^		
American Indian or Alaska Native	0	0
Asian	1 (6)	0
Black	1 (6)	1 (7)
Native Hawaiian or Pacific Islander	0	0
White	13 (81)	12 (86)
Multiracial	1 (6)	0
Decline to answer	0	1 (7)
Ethnicity		
Hispanic	0	1 (7)
Non-Hispanic	16 (100)	13 (93)
Profession		
Physician	12 (75)	NA
APP (PA or NP)	4 (25)	NA
Nurse	NA	3 (21)
Scheduler	NA	4 (29)
Administrative assistant	NA	5 (36)
Medical assistants	NA	2 (14)
Years since training completed, No. (range)	7 (3-16)	NA
Years worked at current hospital, No. (range)	6 (2.5-13)	5 (2-16)
Years worked in current position, No. (range)	6 (2.5-13)	2 (1-4)
EVARs performed or patients interacted with per year		
<5	2 (13)	1 (7)
5-20	8 (50)	2 (21)
>20	5 (31)	8 (57)
Missing Data	1 (6)	2 (14)

Abbreviations: APP, advanced practice provider; EVAR, endovascular aortic repair; HCP, health care professional; NA, not available; NP, nurse practitioner; PA, physician associate.

^a^ Self-reported demographic data collected during a telephone survey conducted immediately following semistructured interviews.

^b^ Percentages may not total 100 due to rounding.

^c^ Race and ethnicity were collected to assess the diversity of the included HCPs and key clinical personnel, in line with purposive sampling techniques.^17^ Race categories, including the multiracial category, were selected and offered to participants for self-selection according to National Institute (NIH) reporting standards.^18^

Of 80 patients contacted, 25 agreed to participate. Thirteen patients (52%) completed follow-up and had a median (IQR) age of 71.6 (63.8-75.6) years, 12 (48%) had an incomplete follow-up and median (IQR) age of 72.0 (66.3-79.3) years. Reasons for declining study participation are listed in eTable 2 in Supplement 1. Of these patients, 17 were male (68%) and 8 were female (32%). All were non-Hispanic individuals, 3 were Black (12%), 20 were White (80%), and 2 declined to answer (8%). Three patients (12%) underwent emergency EVAR for ruptured AAA (2 with complete follow-up, 1 with incomplete follow-up) and 5 (20%) experienced postoperative complications (4 with complete follow-up, 1 with incomplete follow-up). The majority were retired (19 [76%]) and 21 achieved a high school diploma or higher (84%) (Table 2).

**Table 2.** Patient Demographics

Characteristic	Patients, No. (%)^a^	
	Complete follow-up (n = 13)	Incomplete follow-up (n = 12)
Age, y^b^		
>75	6 (46)	6 (50)
Median (IQR)^b^	71.6 (63.8-75.6)	72.0 (66.3-79.3)
Sex		
Female^b^	5 (38)	3 (25)
Male	8 (62)	9 (75)
Race^b^^,^^c^		
American Indian or Alaska Native	0	0
Asian	0	0
Black	2 (15)	1 (8)
Native Hawaiian or Pacific Islander	0	0
White	11 (85)	9 (75)
Decline to answer	0	2 (17)
Ethnicity^b^		
Hispanic	0	0
Non-Hispanic	13 (100)	12 (100)
Rupture^b^	2 (15)	1 (8)
Complication^b^^,^^d^	4 (31)	1 (8)
Travel distance >30 mi^b^	5 (38)	4 (33)
Employment^e^		
Full-time	1 (8)	0
Part-time	0	0
Unemployed	0	0
Self employed	2 (15)	1 (8)
Homemaker	0	0
Student	0	0
Retired	10 (77)	9 (75)
Declined to answer	0	2 (17)
Annual income, $^e^		
<20 000	1 (8)	4 (33)
21 000-60 000	4 (31)	3 (25)
>60 000	7 (54)	2 (17)
Declined to answer	1 (8)	3 (25)
Highest education level^e^		
<High school diploma	0	1 (8)
High school diploma or equivalent	8 (62)	5 (42)
Some college or associate’s degree	4 (31)	3 (25)
Bachelor’s degree	0	0
≥Master’s degree	0	1 (8)
Declined to answer	1 (8)	2 (17)
Marital status^e^		
Unmarried	1 (8)	1 (8)
Married	10 (77)	7 (58)
Divorced	0	2 (17)
Separated	1 (8)	0
Widowed	1 (8)	1 (8)
Declined to answer	0	1 (8)

^a^ Percentages may not total 100 due to rounding.

^b^ Demographic data collected from the electronic health record.

^c^ Race and ethnicity were assessed to ensure the diversity of the included patients was representative of the patient population served by the multisite academic health system, in line with purposive sampling techniques.^17^ Patient race categories were predefined by the institutional electronic health record.

^d^ Complications refer to events occurring within 30 days that include but are not limited to readmission; cardiopulmonary, ischemic, or graft-related adverse events, new renal insufficiency; bleeding or hematoma; or unplanned return to the operating room.

^e^ Self-reported demographic data collected during a phone survey conducted immediately following semi-structured interviews.

Four themes emerged during thematic analysis (Table 3). Factors from the knowledge domain facilitated follow-up, while barriers to follow-up were noted within memory, attention, and decision processes domains. Beliefs about the consequences, reinforcements, and environmental context and resources domains contextually served as both barriers and facilitators.

**Table 3.** Major Themes with Representative Quotes

Theme	Representative quote from HCPs and/or key clinical personnel	Representative quote from patients
1. Patient perceptions of follow-up importance were primarily associated with beliefs about immediate consequences and physical reinforcements; patients who recognized that problems may not have symptoms were more likely to follow-up as directed.	“Well, it’s not as easy to have them [patients] being receptive of the follow-up because everyone is focusing on the immediate success, everybody is focusing on getting out of the hospital alive, and they care less about the follow-up. So yeah, they are not as receptive as they are for the immediate outcomes.” —HCP 01 “…So [EVAR is] shifting the patient’s perspective into this is minimally invasive, this is no big deal. I’m just going to get this stent placed through pokes in my groin and then I’m done. So, I think in a way that perpetuates the belief that it’s not a big deal and again it helps and can push the patients into, you know, validating their idea that they don’t need follow-up.” —HCP 15 “…they [patients] say okay I just traveled all the way to [City] to see the doctor for 10 minutes to tell me everything is okay I feel good I don’t have any pain, this is ridiculous.” —HCP 02 “I think the patients know the initial follow-up is the need to follow-up right after surgery, and that it’s important. I know that they hear that they have to follow-up with us on a regular basis, I’m just not sure how much it actually gets absorbed during the anxiety of facing surgery.” —Key clinical personnel 13	“Some guys have stents put in different places and they don’t go back for a long time; 1 follow-up visit that’s it. They go 5, 10 years without going back…because the doctor doesn’t need to see them all the time.” —Patient 10 (incomplete follow-up) “…they said everything was okay. That was that.…It was to be expected but everything worked out complete. No pain, no problems.” —Patient 04 (incomplete follow-up) “…and I’ve been worried about stroke possibilities because of the operation and so on…but I’ve not had anything like that. I take medicine for it and I’m confident that I should be alright. I don’t think my risk is that high.” —Patient 08 (incomplete follow-up) “…I mean, as a person you have no way of knowing what it looks like inside there or anything. You know, they tell you what they’re going to do, and they tell you what they did, but I didn’t know…without the follow-ups and them checking on things. You just don’t know…if you don’t go for follow-up, who knows if it even adhered or if it worked?” —Patient 11 (complete follow-up) “I believe that if something was wrong, then you know I’m not going to know…that’s why I’m doing it, yeah. You know, because it’s not something that has any symptoms you know, it’s something they have to see so in that sense the only way to make sure everything is going well is to do this by the book.” —Patient 23 (complete follow-up) “If there’s anything wrong, I hope [the doctor] finds it….It’s possible you could have another one occur for one thing, or maybe something starts going wrong with the one they repaired, you never know.” —Patient 08 (complete follow-up)
2. Despite attempts at education by HCPs, patients’ knowledge of anatomical concepts did not equate to better follow-up; however, explicit knowledge of clinical consequences, such as rupture and death did.	“The concept that you can rupture again…I try to explain that to the patients as well. Like I said I use the plumbing analogies and stuff to tell them that things shift, and this stent is actually rendered useless as it shifts and then you can rupture all over again, so I try to emphasize that as well.” —HCP 15 “Most of them are lacking complete understanding. I think they listen to what their doctors speak and sometimes they just say okay, instead of having a clear message. I think medical terminology for a lot of the patients are not understood.” —Key clinical personnel 04 “I think one of the most important from my perspective is if their symptoms are improving or if they have a complication from the surgery, like bleeding open incisions, or if the symptoms are getting worse. This kind of stuff…is the most important after this kind of procedure.” —Key clinical personnel 14	“Yeah, it’s to make sure there ain’t no [unintelligable] around the graft being in place, make sure it is staying in place and make sure that all my vascular are working, you know, like the flow to my feet, the flow to my legs, my arms my chest. You know that’s your main artery.” —Patient 06 (incomplete follow-up) “I assumed they were looking at the positioning of the stent. And I guess the movement of the blood through the aneurysm or what was left of it…I guess that there was some, not interference, but the renal arteries were involved I guess, and the one, and that of course is accomplished by the construction of the stent.” —Patient 20 (incomplete follow-up) “They told me I’d have to check to see if the aneurysm is stabilized, that the stent didn’t move.” —Patient 21 (incomplete follow-up) “When asked about the potential benefits of following up, a patient said ‘Staying alive!’ I think the continuing follow-ups for the rest of my life I think was told to me the first visit after…” —Patient 09 (complete follow-up) “You’re not just walking blindly thinking ‘What if my aneurysm bursts open?’ You know? I think at least you know it’s being checked. And I’m not saying that something couldn’t still happen, because of course it can at any time, but at least that makes you not worry as much, I think. I don’t know how else to put it, but it takes some of that fear away.” —Patient 11 (complete follow-up) “Make sure everything’s alright…and that nothing grows anymore!” —Patient 22 (complete follow-up)
3. Patient memory and attention issues were a barrier to follow-up. In addition to patient-initiated attention aids, this was partially mitigated by protocolization of follow-up processes (eg, appointment reminders, standardization of preoperative and postoperative resources, and optimization of discharge paperwork).	“Many of these patients, I mean remember these are older patients, they got pneumonia [then] they got admitted to a hospital, [then] they got sick [then] they get sent to rehab [then] they have a stroke, [then] they have a family member who dies or get cancer, [then] they lose a spouse. So, the reasons why they kind of lose track of what they’re supposed to do is not simply the fact that they don’t want to come, or they don’t take ownership, or they are not interested, there’s always a lot of reasons.” —HCP 02 “The discharge instructions are like 20 pages long. They go through everything. So, somewhere in that mix I know there’s information on what an EVAR is and what they did and what we did but there is so much jam packed into those discharge instructions that I think it gets lost.” —Key clinical personnel 01	“I didn’t have a follow up meeting because we had heavy snow at the time it was scheduled…and I couldn’t keep that appointment, I had cancel it. And at the time I said I would consider it in [month], and [month] came and I forgot about it because of this COVID thing coming up, you know, and I forgot about it so I didn’t have an examination after the surgery.” —Patient 02 (incomplete follow-up) “Well, I don’t know for sure [about follow-up] because I have other medical issues going on right now that it just is as important as that if not more so.” —Patient 05 (incomplete follow-up) “I don’t even remember which one [surgery] I had….As far as [following up], I can’t recall. I know I probably didn’t go back….But a lot of things I don’t care about I don’t remember them.” —Patient 16 (incomplete follow-up) “The [hospital system application] is perfect because it sends me reminders about a week before that I have an appointment coming up, and it all gets done through my app…I even thank them because sometimes my appointments are made a year in advance, and if it wasn’t for that app, I would probably forget about the appointment.” —Patient 11 (complete follow-up) “Oh, it’s easy, I have it marked on my calendar…they send me a message on my cell phone that I have an appointment coming up.” —Patient 12 (complete follow-up) “No, he has so many different doctors you know we just keep everything in our calendar book, he’s laughing about it, we keep everything in our calendar book, and we just use it as a calendar.” —Caretaker (wife) of Patient 15 (complete follow-up)
4. Social support and environmental circumstances, particularly transportation, had implications for follow-up. Telemedicine might also be helpful, but its widespread use has limitations.	“…A concerned wife is basically like 100% predictor of good follow up.” —HCP 04 “…and so, you know, we often get those ultrasounds [in the community] and they’re sort of useless, now. Yeah, it’s easy you can get CT scans professionally but that comes with the risk of radiation exposure and so on, which is not needed.” —HCP 08 “Schedulers and secretaries often take it on themselves to handle difficult situations. There are times when patients call and say I’m in a wheelchair I need someone to pick me up at the front door…we take a lot of it on ourselves and try to navigate their issues….Those resources you kind of create and build lists within your departments yourself because…they’re not all found in 1 place.” —Key clinical personnel 03	“Yes like I told him at [hospital system], I cannot go up there as often as they would like me to for follow-ups simply because my brother-in-law takes me, my brother-in-law is [8X] years old, I’m [7X], his wife just recently passed away, which is my sister, and it’s very hard on him. And I won’t get on one of those buses because I don’t know anybody, and I very seldom to go out of my home because of this COVID.” —Patient 06 (incomplete follow-up) “I live alone, there’s a lot of cons with that….If I have to go to [City] it can be a challenge…[City] is a long way, and I have to depend on someone else to get me there because they won’t let me drive myself. I think the biggest benefit would be to try to get the appointment closer to my home, but I know that’s not possible with the surgeon.” —Patient 14 (incomplete follow-up) “I’m not very technical you know, I’m not very computer oriented plus I just don’t like the idea I’d rather see them personally.” —Patient 23 (incomplete follow-up) “I’m able to drive now, so that that takes a load off. Before then if I couldn’t, my wife would either drive me or my sister, so I have superior support.” —Patient 03 (complete follow-up) “What I have to do I don’t remember…Yeah, my wife had to tell me that [follow-up was needed] and then I did it and whatever. I mean, whatever she says I have to do I’ve got to do it.” —Patient 17 (complete follow-up) “Well, if I had to [follow-up via telemedicine], but it’s nice always seeing our doctor…Plus I have to go to the, what is it? Sonogram, I have to do a sonogram first…and then the [doctor]’s gonna tell me all about it.” —Patient 22 (complete follow-up)

Abbreviations: CT, computed tomography; EVAR, endovascular aortic repair; HCP, health care professional.

### Theme 1: Beliefs About Consequences and Physical Reinforcements

Patient perceptions of follow-up importance were primarily affected by beliefs about immediate consequences and physical reinforcements. Patients who recognized that problems may not have symptoms were more likely to follow-up as directed.

Most HCPs emphasized long-term follow-up strategies and consequences of nonadherence in preoperative conversations, admitting that some HCPs are more skilled at these conversations than others. HCPs believed initial anxiety over immediate consequences takes precedence for patients and may cloud their receptivity during these discussions. Several patients mentioned feeling scared or overwhelmed simply hearing the word *aneurysm*. One patient noted “among everything else, it [follow-up] gets lost.”

Postoperatively, many HCPs described the minimally invasive nature of EVAR and lack of pain or large incisions as a negative reinforcement (deterrent) for follow-up. Postoperatively, patients reported the initial fear surrounding the operation quickly subsided. Most experienced rapid recovery, perpetuating a focus on immediate outcomes like discharge and return to daily life rather than future complications. This contributed to a notion, particularly among patients with nonadherence to follow-up, that EVAR follow-up was “no big deal.” Key clinical personnel similarly emphasized the immediate outcomes postoperatively and were infrequently able to communicate long-term consequences of follow-up nonadherence. There was also misconception among key clinical personnel and patients that complications will cause symptoms, particularly among patients with incomplete follow-up patterns. Many patients reported reliance on pain to prompt return visits, but without it, there exists an attitude of “no pain, no problems.”

Patients with complete follow-up, however, were more likely to recognize that adverse outcomes associated with EVAR were not necessarily immediate, and frequently asymptomatic. These patients emphasized the preventative nature of follow-up and imaging, and the role of the medical team (rather than physical reinforcements alone) in detecting delayed complications.

### Theme 2: Knowledge of Anatomical Concepts vs Clinical Consequences

Despite attempts by HCPs to provide education, patients’ knowledge of anatomical concepts did not translate to better follow-up; however, explicit knowledge of clinical consequences, such as rupture and death, did. Many HCPs described using visual aids and analogies to explain anatomy of EVAR failures to patients to encourage follow-up. Yet, HCPs described time constraints as a contributing factor in inadequate patient education. Time limitations were reiterated by key clinical personnel as well as fear of being penalized for advising above their clinical role; therefore, most key clinical personnel deferred patient questions to HCPs. Additionally, many key clinical personnel and HCPs felt anatomical information, especially when not presented in plain language, was too abstract for some patients and did not necessarily contribute to their understanding that lack of follow-up can be life-threatening. Indeed, we found no association between patient knowledge of anatomy or mechanisms of failure and patient follow-up patterns in our study. Several patients misconstrued EVAR as a stent similar to other stents they or others have had which may not require the same type of follow-up scheme.

Patients’ beliefs about consequences of incomplete follow-up, such as risk of life-limiting complications, appeared to motivate follow-up. Several HCPs and key clinical personnel talked about the importance of deliberately using scare tactics by explaining to patients that follow-up nonadherence can result in aneurysm rupture and death. Several patients with complete follow-up explicitly stated they were following up to prevent future rupture.

### Theme 3: The Role of Memory and Attention Issues in EVAR Follow-Up

Patient memory and attention issues were a barrier to follow-up. In addition to patient-initiated attention aids, this barrier was partially mitigated by protocolization of follow-up processes (appointment reminders, standardization of preoperative and postoperative resources, optimization of discharge paperwork, and so on).

Both HCPs and key clinical personnel believed patients often intended to follow-up but forget due to the overwhelming amount of information presented at initial consultations. Patients, particularly those older than 75 years, frequently reported juggling multiple medical problems and family issues. EVAR follow-up thus may be a lesser priority.

Many HCPs emphasized trying to overcome patient attention and memory issues with repetition. Several mentioned use or potential use of verbal or written contracts to reinforce follow-up importance. There was some disagreement among HCPs and key clinical personnel regarding the utility of handouts and discharge paperwork combatting memory and attention issues. Several described that more streamlined versions of both informational packets and discharge paperwork were needed given that current versions are cumbersome, with follow-up frequently getting lost in a stack of unhelpful documents.

Many patients pointed out that given the long length of time between visits, they did not necessarily remember specific follow-up recommendations but nevertheless reported they did what they were told. All participants expressed that communication in multiple forms, including letters, telephone calls, emails, and notifications from the health care system application, helped remind patients of appointments. Patients younger than 75 years were more likely to use the health care system application or email. Infrequently, key clinical personnel personally contacted patients after missed or no-show appointments, which was sometimes successful in bringing patients in if they simply had forgotten. Some HCPs reported having a personal list that they or key clinical personnel use to monitor follow-ups. Ultimately, many HCPs and key clinical personnel advocated for a more robust registry to call back patients who missed their appointment but acknowledged that this is time-consuming and costly.

### Theme 4: Implications of Social Support and Environmental Circumstances

Social support and environmental circumstances, particularly transportation, were believed to have substantial implications for follow-up. Participants believed that telemedicine could be helpful, but widespread use had limitations.

Many HCPs, key clinical personnel, and patients with complete follow-up described having family support to remember appointment times, take notes, and assist in care coordination. Several patients also described having long-term relationships with their PCPs, conferring a singular source of trusted information, particularly if the PCP was within the same health system as the surgeon. Patients with incomplete follow-up often described disjointed or nonexistent support networks. Several patients also described having roles as caretakers for family members, and in-turn, neglected their own health.

Respondents from all groups felt transportation was a substantial barrier to follow-up. Many patients relied upon family to drive them or public transportation. The walk from the parking lot to the office was long and confusing. Patients must pay for parking and gas. Resources to arrange patient rides were inconsistently available and only shared through word of mouth among key clinical personnel rather than through a centralized system. Furthermore, several key clinical personnel described the difficulty of scheduling imaging and follow-up on the same day due to staffing issues at both clinics and imaging centers, resulting in multiple visits to coordinate.

Several solutions to transportation and costs were offered. Many participants suggested free parking or hospital-sponsored transportation as is present in the Veterans’ Affairs health system. Other respondents suggested that HCPs visit sites remote from major health systems, closer to patients. For out-of-network patients, some HCPs described arranging alternative follow-up with in-network HCPs. One key clinical person described involving social workers for patients with complicated needs.

HCPs generally agreed that telemedicine could ameliorate transportation issues in the future, but current limitations exist. While most HCPs expressed willingness to use telemedicine to review locally obtained computed tomography (CT) scans, they were hesitant to rely on outside hospital ultrasonography given the high degree of operator dependency. Telemedicine may also be limited by equipment, level of patient comfort with technology, or consistent internet access for some rural areas or patients living in socioeconomic disadvantaged areas. Lack of information technology support services or training on the use of a telemedicine application further limited regular telemedicine use. Although telemedicine was often used during the COVID-19 pandemic, many patients, regardless of age, preferred face-to-face interactions, particularly if they did not have an established relationship with a vascular surgeon.

## Discussion

Our qualitative evaluation of factors associated with patient follow-up after EVAR revealed that patient perceptions of EVAR as well as memory and attention practices were barriers to follow-up adherence. Knowledge of explicit long-term outcomes, robust social support, and protocolization of resource distribution and patient education facilitated follow-up. Potential solutions to improve follow-up included refined protocolization and heightened resources in the preoperative and postoperative periods (ie, recall system, streamlined discharge paperwork, increased use of health care application reminders, widespread knowledge of social support services), as well as transportation adjuncts. Effective patient preoperative education should emphasize the long-term consequences and risks of neglected follow-up, including aneurysm rupture and death rather than complex anatomy. Furthermore, this education should focus on the almost universally asymptomatic nature of complications. Telemedicine can facilitate EVAR surveillance and follow-up in the future; however, implementation is currently limited by patient willingness and comfort using technology as well as the reliability of local ultrasonography, which is highly operator dependent.

Our findings support and contextualize those previously reported in retrospective quantitative studies. Several studies have reported associations between social factors, such as driving distance and lack of a PCP, with follow-up nonadherence, which our study confirms.^6,7^ Previous studies also found that age and presence of medical comorbidities, including hypertension and prior stroke, have adverse implications for follow-up adherence.^7,8,9,10,20^ We also found that patients who manage multiple medical problems and those with cognitive impairment due to advanced age or medical comorbidities struggle to keep up with appointments. Many of these issues, in addition to transportation barriers and memory deficits, can be offset by the presence of strong social support; however, large disparities in patient environment and resources exist. Hospital-sponsored transportation adjuncts, such as the Veterans Transportation Services used in the Veterans’ Affairs health system, may be useful for mitigating transportation-related issues.^21^ Additionally, screening for patients with poor social support at hospital discharge and building a list of standardized transportation services to facilitate follow-up with social work assistance may be beneficial proactive measures to improve EVAR follow-up.

Protocolization, which involves standardized sets of reminders for follow-up (eg, notifications via a health care application, text messages, emails, telephone calls, and/or letters), was suggested by respondents to be beneficial for most participants. Several key clinical personnel and HCPs also called for universal adoption of a recall system to keep track of patients with missed follow-up visits despite existing measures. However, the paperwork provided to patients at both clinic visits and hospital discharge was thought to be nonstandardized and often too long. Paper resources, particularly discharge paperwork, should be refined and tailored to appropriate reading levels and/or older patients with potential visual impairment. Current lack of reliable written resources further places burden on HCPs to continuously restate the importance of follow-up, as well as trusting patients’ memory among the stresses of surgery. Standardization of education and resources for all members of the health care team, particularly key clinical personnel, may also help reinforce appropriate follow-up practices and lessen the burden on HCPs.

Prior survey research has found that few patients had any preexisting knowledge about AAA prior to the initial consultation.^22^ We found that patients often felt so overwhelmed by the amount of information shared at initial consultations that they were unable to absorb the importance of follow-up. Many patients also likened EVAR to cardiac stents that have less stringent follow-up guidelines and often believed any device-related issues would be symptomatic. Some key clinical personnel also believed longer-term postoperative complications would have symptoms. Many HCPs spent substantial time at clinic visits explaining aortic anatomy and endoleak physiology to patients; however, this information was often too abstract for patient-level understanding and did not necessarily translate into better follow-up as intended. However, the rate of follow-up was higher among patients who understood the serious consequences related to follow-up nonadherence, such as aneurysm rupture, death, and long-term complications, and those who understood that complications were frequently asymptomatic.

Telemedicine has the potential to combat many EVAR follow-up issues, particularly those related to transportation and difficulty coordinating same-day imaging and follow-up. However, participants’ perceptions were mixed about the practicality of using telemedicine for this disease process given the older patient population and residence in socioeconomically disadvantaged areas. Feasibility of telemedicine may be ameliorated by family or key clinical personnel support. HCPs were often happy to conduct follow-up using telemedicine for patients with CT scans obtained locally, however, they felt uncomfortable trusting ultrasonography outside of their institution. One meta-analysis suggested that magnetic resonance imaging is more sensitive at endoleak detection than CT angiography and may be another alternative for patients who live far from the treating institution while still avoiding excess radiation.^23^

Finally, emerging data on the clinical importance of long-term surveillance after EVAR remains mixed. Although loss to follow-up has been associated with increased mortality,^6^ a retrospective study of more than 500 patients who underwent EVAR found sac shrinkage to 40 mm or less was associated with decreased risk of rupture or sac reexpansion.^24^ Andraska et al^24^ therefore suggested patients meeting this threshold can safely extend follow-up frequency to 3-year intervals. Furthermore, several studies have found that patients with identified type 2 endoleaks undergo more reinterventions without survival benefit, even in those patients experiencing sac enlargement.^25,26,27^ HCPs should thus carefully consider the implications of surveillance intervals for patient outcomes given these findings and known barriers to EVAR follow-up, and resources to improve follow-up should be deployed judiciously.

This qualitative study presents a novel approach to identifying the nuances of follow-up nonadherence after EVAR from patient and HCP perspectives that was not possible in previous quantitative work. The findings, guided by the TDF, present several areas of targeted intervention to improve follow-up adherence for EVAR. These findings may apply to other surgical patient populations with similar medical complexity who require serial imaging and long-term surveillance, including patients with breast and colon cancer.^28,29^ Furthermore, the robustness of the study’s findings was ensured not only through investigator triangulation (with involvement of multiple study team members with diverse backgrounds at all stages of data collection, consensus coding, and analysis), but also by achieving thematic saturation via comparison of perspectives from multiple participant groups (ie, patients, HCPs, and key clinical personnel).

### Limitations

Regional differences in practice patterns and clinic logistics may limit the transferability of our results. Our patient cohort consisted of primarily non-Hispanic white men, although this is concordant with existing epidemiologic data.^30^ Additionally, we were able to recruit only 3 patients who underwent emergency EVAR (half of our targeted recruitment) because many patients with rupture were deceased at the time of recruitment.^16^ Thus, we were unable to stratify analyses by elective vs emergency repair. Finally, we limited analysis to barriers and facilitators of EVAR follow-up adherence, comprising primarily 5 TDF domains. Future work should explore the implications of remaining domains for EVAR follow-up patterns and interactions among members of the health care team.

## Conclusions

In this qualitative study investigating the barriers and facilitators to patient follow-up after EVAR, patient perceptions of immediate outcomes after EVAR and memory and attention issues posed barriers to follow-up, while recognition of explicit long-term consequences, presence of social support, and protocolization facilitated follow-up. Potential interventions to improve follow-up behaviors included enhanced protocolization, heightened resources in preoperative and postoperative periods, selectively expanded telemedicine, and transportation adjuncts.

## Notes

Supplement 1. **eTable 1.** Qualitative Codebook Used for Thematic Analysis **eTable 2.** Response to Patient Recruitment and Reasons for Declining Study Participation

Supplement 2.
Data Sharing Statement

## References

[1] Salata K, Hussain MA, de Mestral C, . Comparison of outcomes in elective endovascular aortic repair vs open surgical repair of abdominal aortic aneurysms. JAMA Netw Open. 2019;2(7):e196578. doi:10.1001/jamanetworkopen.2019.6578 31290986 PMC6624804

[2] Chaikof EL, Dalman RL, Eskandari MK, . The Society for Vascular Surgery practice guidelines on the care of patients with an abdominal aortic aneurysm. J Vasc Surg. 2018;67(1):2-77.e2. doi:10.1016/j.jvs.2017.10.044 29268916

[3] Melillo AM, Trani JL, Gaughan JP, Carpenter JP, Lombardi JV. Assessing trends, morbidity, and mortality in ruptured abdominal aortic aneurysm repair with 9 years of data from the National Surgical Quality Improvement Program. J Vasc Surg. 2020;71(2):423-431. doi:10.1016/j.jvs.2019.04.462 31227411

[4] Andersson M, Talvitie M, Benson L, Roy J, Roos H, Hultgren R. A population-based study of post-endovascular aortic repair rupture during 15 years. J Vasc Surg. 2021;74(3):701-710.e3. doi:10.1016/j.jvs.2021.01.065 33617983

[5] Hicks CW, Zarkowsky DS, Bostock IC, . Endovascular aneurysm repair patients who are lost to follow-up have worse outcomes. J Vasc Surg. 2017;65(6):1625-1635. doi:10.1016/j.jvs.2016.10.106 28216362 PMC5960980

[6] Phillips AR, Andraska EA, Reitz KM, . Any postoperative surveillance improves survival after endovascular repair of ruptured abdominal aortic aneurysms. Ann Vasc Surg. 2022;80:50-59. doi:10.1016/j.avsg.2021.09.047 34775012 PMC8897248

[7] Wu CY, Chen H, Gallagher KA, Eliason JL, Rectenwald JE, Coleman DM. Predictors of compliance with surveillance after endovascular aneurysm repair and comparative survival outcomes. J Vasc Surg. 2015;62(1):27-35. doi:10.1016/j.jvs.2015.02.023 25864044

[8] Shiraev TP, Durur E, Robinson DA. Factors predicting noncompliance with follow-up after endovascular aneurysm repair. Ann Vasc Surg. 2018;52:30-35. doi:10.1016/j.avsg.2018.03.037 29793019

[9] Schanzer A, Messina LM, Ghosh K, . Follow-up compliance after endovascular abdominal aortic aneurysm repair in Medicare beneficiaries. J Vasc Surg. 2015;61(1):16-22.e1. doi:10.1016/j.jvs.2014.06.006 25441010 PMC4276501

[10] Garg T, Baker LC, Mell MW. Adherence to postoperative surveillance guidelines after endovascular aortic aneurysm repair among Medicare beneficiaries. J Vasc Surg. 2015;61(1):23-27. doi:10.1016/j.jvs.2014.07.003 25088738

[11] Atkins L, Francis J, Islam R, . A guide to using the Theoretical Domains Framework of behaviour change to investigate implementation problems. Implement Sci. 2017;12(1):77. doi:10.1186/s13012-017-0605-9 28637486 PMC5480145

[12] Michie S, van Stralen MM, West R. The behaviour change wheel: a new method for characterising and designing behaviour change interventions. Implement Sci. 2011;6:42. doi:10.1186/1748-5908-6-42 21513547 PMC3096582

[13] Patey AM, Islam R, Francis JJ, Bryson GL, Grimshaw JM; Canada PRIME Plus Team. Anesthesiologists’ and surgeons’ perceptions about routine pre-operative testing in low-risk patients: application of the Theoretical Domains Framework (TDF) to identify factors that influence physicians’ decisions to order pre-operative tests. Implement Sci. 2012;7:52. doi:10.1186/1748-5908-7-52 22682612 PMC3522997

[14] Pearse BL, Keogh S, Rickard CM, . Bleeding management practices of Australian cardiac surgeons, anesthesiologists and perfusionists: a cross-sectional national survey incorporating the Theoretical Domains Framework (TDF) and COM-B Model. J Multidiscip Healthc. 2020;13:27-41. doi:10.2147/JMDH.S232888 32021232 PMC6970603

[15] Tong A, Sainsbury P, Craig J. Consolidated criteria for reporting qualitative research (COREQ): a 32-item checklist for interviews and focus groups. Int J Qual Health Care. 2007;19(6):349-357. doi:10.1093/intqhc/mzm042 17872937

[16] Phillips AR, Olivere LA, Jarosinski MC, . Identifying barriers and facilitators to follow-up after endovascular aortic repair (EVAR): qualitative study design and protocol. MethodsX. 2024;13:102938. doi:10.1016/j.mex.2024.102938 39286439 PMC11403246

[17] Palinkas LA, Horwitz SM, Green CA, Wisdom JP, Duan N, Hoagwood K. Purposeful sampling for qualitative data collection and analysis in mixed method implementation research. Adm Policy Ment Health. 2015;42(5):533-544. doi:10.1007/s10488-013-0528-y 24193818 PMC4012002

[18] Revision: NIH policy and guidelines on the inclusion of women and minorities as subjects in clinical research. National Institutes of Health. Updated July 17, 2025. Accessed September 26, 2025. https://grants.nih.gov/grants/guide/notice-files/NOT-OD-25-131.html

[19] Guest G, Namey E, Chen M. A simple method to assess and report thematic saturation in qualitative research. PLoS One. 2020;15(5):e0232076. doi:10.1371/journal.pone.0232076 32369511 PMC7200005

[20] Wolf S, Ashouri Y, Succar B, . Follow-up compliance in patients undergoing abdominal aortic aneurysm repair at Veterans Affairs hospitals. J Vasc Surg. 2024;80(1):89-95. doi:10.1016/j.jvs.2024.02.040 38462060

[21] Health benefits: Veterans Transportation Program (VTP). U.S. Department of Veterans Affairs. Updated December 4, 2024. Accessed January 24, 2025. https://www.va.gov/healthbenefits/vtp/

[22] Anderson PB, Wanken ZJ, Perri JL, ; PROVE-AAA Study Team. Patient information sources when facing repair of abdominal aortic aneurysm. J Vasc Surg. 2020;71(2):497-504. doi:10.1016/j.jvs.2019.04.460 31353272 PMC10767985

[23] Habets J, Zandvoort HJ, Reitsma JB, . Magnetic resonance imaging is more sensitive than computed tomography angiography for the detection of endoleaks after endovascular abdominal aortic aneurysm repair: a systematic review. Eur J Vasc Endovasc Surg. 2013;45(4):340-350. doi:10.1016/j.ejvs.2012.12.014 23403221

[24] Andraska EA, Phillips AR, Reitz KM, . Longer follow-up intervals following endovascular aortic aneurysm repair are safe and appropriate after marked aneurysm sac regression. J Vasc Surg. 2022;76(2):454-460. doi:10.1016/j.jvs.2022.01.079 35093463 PMC9329192

[25] Mulay S, Geraedts ACM, Koelemay MJW, Balm R; ODYSSEUS study group. Type 2 endoleak with or without intervention and survival after endovascular aneurysm repair. Eur J Vasc Endovasc Surg. 2021;61(5):779-786. doi:10.1016/j.ejvs.2021.01.017 33632609

[26] Mansukhani NA, Brown KR, Zheng X, Mao J, Goodney PP, Hoel AW. High incidence of type 2 endoleak and low associated adverse events in the Vascular Quality Initiative linked to Medicare claims. J Vasc Surg. 2023;78(2):351-361. doi:10.1016/j.jvs.2023.04.013 37086823 PMC10524631

[27] Cifuentes S, Rodrigues DVS, Tabiei A, . Implications and late outcomes of persistent type II endoleaks after endovascular aneurysm repair. J Vasc Surg. 2023;78(3):e13-e14. doi:10.1016/j.jvs.2023.06.028 38663777

[28] Brauer ER, Long EF, Petersen L, Ganz PA. Current practice patterns and gaps in guideline-concordant breast cancer survivorship care. J Cancer Surviv. 2023;17(3):906-915. doi:10.1007/s11764-021-01152-1 34970715 PMC9243187

[29] Mollica MA, Mayer DK, Oeffinger KC, . Follow-up care for breast and colorectal cancer across the globe: survey findings from 27 countries. JCO Glob Oncol. 2020;6:1394-1411. doi:10.1200/GO.20.00180 32955943 PMC7529533

[30] Li SR, Reitz KM, Kennedy J, . Epidemiology of age-, sex-, and race-specific hospitalizations for abdominal aortic aneurysms highlights gaps in current screening recommendations. J Vasc Surg. 2022;76(5):1216-1226.e4. doi:10.1016/j.jvs.2022.02.058 35278654 PMC9458770

