# Supplementary material for: Barriers and Facilitators to Endovascular Aortic Repair Follow-Up
Source: JAMA Netw Open. 2025 Dec 2;8(12):e2546327. doi: 10.1001/jamanetworkopen.2025.46327 (PMC12673408; doi:10.1001/jamanetworkopen.2025.46327)
Supplement: Supplement 1. — eTable 1. Qualitative Codebook Used for Thematic Analysis eTable 2. Response to Patient Recruitment and Reasons for Declining Study Participation [file jamanetwopen-e2546327-s001.pdf]

Supplemental Online Content

Jarosinski MC, Olivere LA, Barnes JL, Tzeng E, Liang NL, Rak KJ, Philips AR. Examining barriers and facilitators to endovascular aortic repair follow-up. *JAMA Netw Open.*2025;8(12): e2546327. doi:10.1001/jamanetworkopen.2025.46327

eTable 1. Qualitative Codebook Used for Thematic Analysis
eTable 2. Response to Patient Recruitment and Reasons for Declining Study Participation

This supplemental material has been provided by the authors to give readers additional information about their work.

© 2025 Jarosinski MC et al. *JAMA Network Open.*

eTable 1: Qualitative Codebook Used for Thematic Analysis*

1° code	2° code	3° code	4° code	*Definitions*	Inclusions	Exclusions
**Knowledge related to follow up**				All aspects related to background knowledge related to EVAR as well as the need for follow-up after EVAR, including what is provided to the patient and how it is received.	Focus on follow-up and impacts for follow-up	
	HCP			Follow-up EVAR-related knowledge that HCP have as well as give to patients including aspects of how they provide the information to patients		Providers other than surgeons, fellows, residents involved in vascular
		self		HCP's personal knowledge of follow-up needed		
		given to patient		What information HCP provide to patients (and family) related to the need for follow-up		
			visual aids used	Use of pictures, diagrams, or models to explain the anatomy or procedure of an EVAR	Included use of imagery "picture a turtleneck with sleeves cut off"	
	Key Clinical Personnel			Follow-up EVAR-related knowledge that KP have as well as give to patients including aspects of how they provide the information to patients		
		self		KP's personal knowledge of follow-up needed		
		given to patient		What information KP provide to patients (and family) related to the need for follow-up		
	Patient			What patients know about follow up and the need for follow-up	Time and dates of upcoming follow-up	
**Beliefs about Consequences**				Participant's description of consequences of follow up strategy	Statements from the perspective of the person being interviewed	Capture beliefs about consequences from HCP's perspective as "Knowledge'
	No consequences			Statements that there are no consequences, or lack or consequences, for follow-up strategy		

© 2025 Jarosinski MC et al. *JAMA Network Open.*

1° code	2° code	3° code	4° code	Definitions	Inclusions	Exclusions
	Consequences			Descriptions of consequences that may happen based on follow up choices		
		Immediate (post-op surgical site healing)		Description of immediate or short-term consequences of EVAR (surgical healing)		
		Death		Description of death as a consequence of EVAR follow-up		
		Long-term consequences (another aneurysm)		Description of long term health implications, like stent movement or another aneurysm, as a result of EVAR follow-up	Specific description of event, or "lifelong" descriptors	
**Reinforcements**				Descriptions of various relationships or reinforcements present in the follow up process, both physical and social		
	Healthcare given			Relationships or reinforcements that are given by the healthcare team, like a good follow-up experience, or reminders about importance of follow-up		
		Expectation setting		Descriptions of initial communications about expected or required follow-up after EVAR, (usually) preoperative with surgeon		Limit to preop (usually) conversations had with surgeon
		Messaging about need for follow up		Descriptions of communicating about the importance or value of follow-up after EVAR		Mentions responsibility for messaging - code as social/ professional role. Include only descriptions of the moment of messaging.
		patient contact		Instances of patient contact or touch points outside of scheduled follow up visits (e.g. a card, letter, patient initiated phone call)		

© 2025 Jarosinski MC et al. *JAMA Network Open.*

1° code	2° code	3° code	4° code	Definitions	Inclusions	Exclusions
		Healthcare team relationships or rapport		Descriptions of positive or negative interactions with health care team that impact patients' decisions related to follow-up	Descriptions of a specific provider relationships rather than healthcare writ large	
		Scare patients/ dire consequences		When HCP describe that they use worst-case or most extreme negatives when providing information about follow-up to stress the importance		
	Visit experience			Descriptions of doctors visit experiences		
		Vascular clinic visit experiences		Descriptions of preoperative or follow-up visit experiences with vascular physicians that include potential reinforcer		
		Vascular inpatient surgical experiences		Vascular surgical or inpatient experience		
	Physical reinforcements or pain			Descriptions of physical pain or similar indications (or lack of these indications) that follow-up is needed	scared by near death experiences?	
	Complication			(leak, etc, that prompted action related to follow-up)		

© 2025 Jarosinski MC et al. *JAMA Network Open.*

1° code	2° code	3° code	4° code	Definitions	Inclusions	Exclusions
**Memory, Attention, and Decision Processes**				Descriptions of memory or attention factors that impact decision making processes or ability to follow-up or support follow-up		
	HCP			Descriptions of memory or attention factors that impact the healthcare team's decisions or ability to support follow-up		
	Key Clinical Personnel			Descriptions of memory or attention factors that impact the healthcare team's decisions or ability to support follow-up		
	Patient			Descriptions of memory or attention factors that impact decision making processes or ability to follow-up	"I don't remember" comments in parent code	
		Other health conditions		Descriptions of a patient being impacted or overwhelmed by multiple medical issues in a way that it affects ability to prioritize follow-up		Patient does not describe it as impactful of attention/ decisions
		Note taking		Descriptions of physical notes taken by patient to aid follow-up		Notes taken by someone other than patient (should be social support)
		Calendar		Descriptions of use of a calendar to aid follow-up memory and attendance		
	Healthcare application			Descriptions of use of the healthcare application		
**Environmental Context and Resources**				*Elements of the environment and resources around participants that may impact follow-up or support of follow-up*		
	HCP			Elements of the environment and resources around HCP participants that may impact follow-up or support of follow-up	info resources, workplace environmental context	

© 2025 Jarosinski MC et al. *JAMA Network Open.*

1° code	2° code	3° code	4° code	Definitions	Inclusions	Exclusions
	Key Clinical Personnel			Available resources that providers and KPs might access in order to support follow-up	info resources, workplace environmental context	
	Patient			Elements of the patient's environment, including social environment and resources that are available to them		
		Transportation		Descriptions of transportation resources or lack of transportation available in environment		
			Distance	Description of distance as contributing to patient's follow-up experience		
		Financial		Description of financial factors contributing to patient's follow-up experience		
		Other responsibilities/ time		Description of lack of time or having time as contributing to patient's follow-up ability. Or, other patient responsibilities contributing to follow-up ability		
		social support		Descriptions of loved ones, care workers, or other social support available to patient, or lack of social support in patient's life	Co-code when used as a memory aid	Elements of social support that are more persuasive or cognitive - may belong under social influence
		Informational resources - Patient		Any available resources that patients might access in order to learn more about the need for follow-up	Include paper reminders of when to make appointment (Depart), website information	
			Depart/ discharge paperwork	Information given to the patient within the Depart paperwork upon discharge	co-code with areas of identified improvement process when applicable	

© 2025 Jarosinski MC et al. *JAMA Network Open.*

1° code	2° code	3° code	4° code	Definitions	Inclusions	Exclusions
**Telemedicine**				Comments related to the offering or use of telemedicine for EVAR follow-ups, including whether it might be utilized hypothetically or in future		Facilitator/ barriers - keep to follow-up in general
	When offered			Providers' comments on whether and when they offer telemedicine options for EVAR follow-up visits		
	Acceptability			Comments on the value or opinion of using telemedicine as an option for EVAR follow-up visits		
	Feasibility			*Comments on logistics of using telemedicine options for EVAR follow-up visits*		
		IT		*Comments on patients' access to devices or internet for telemedicine use*		
**Identified areas of improvement**				Descriptions of tangible needs for improvements related to the follow-up process	Co-code where applicable	Non-tangible descriptions
	self			Descriptions of examples for improvement or needs related to participant's own role		
	process			Descriptions of examples for improvement or needs related to the follow-up process		Code improvements to participant's role in the process in "self"
**Facilitators**				Things that may promote follow-up and may include aspects or patterns from historical practices	Descriptions of a lack of barrier. Possible co-coding	
**Barriers**				Things that may discourage follow-up and may include aspects or patterns from historical practices	Possible co-coding; may include breakdowns in process	

© 2025 Jarosinski MC et al. *JAMA Network Open.*

1° code	2° code	3° code	4° code	Definitions	Inclusions	Exclusions
**Process**				Descriptions of various parts of the process of establishing EVAR follow-up		
	Preop conversations /giving information			Comments about pre-op conversations before surgery related to expectations or shared information about follow-up		
	Surgery			Comments on the surgery experience or inpatient experience		
	Discharge			Comments related to the patient discharge experience		
	Arranging follow-up			Comments related to the process of arranging follow-up, including long term arrangement of follow-up for both groups (post 1 month)		
		Non-rupture (planned)		Descriptions of the typical process of arranging follow-up (in most cases, planned, non-emergent)		
		Rupture (emergent)		Descriptions of arranging follow-up for rupture or emergent patients		
	Follow-up Imaging			Imaging related to attending follow-up		
		Local imaging		Description of maging (CT scanor US) performed at local health system, distinct from quaternary health system where EVAR performed		

© 2025 Jarosinski MC et al. *JAMA Network Open.*

1° code	2° code	3° code	4° code	Definitions	Inclusions	Exclusions
	Attending follow-up			Experiences related to clinic follow up including comments on imaging or even generalized comments about follow-up without disentangling clinical visit and imaging	Comments on the clinic visit itself and related imaging	Mentions of transportation to and from follow up - code transportation, descriptions of factors that make follow up less/more likely (code in patient factors)
	When missed/ canceled			Descriptions of processes that occur when a follow-up appointment is missed or canceled	Including descriptions of patients missed or canceled appointment	
	Interval questions					
**COVID-19**				Any factors related to follow-up that were impacted or influenced by COVID-19		

*Abbreviated codebook with primary (1°), secondary (2°), tertiary (3°), and quaternary (4°) codes for themes discussed in this manuscript related to 5 Theoretical Domains Framework domains: knowledge; memory, attention, and decision processes; beliefs about consequences; environmental context and resources; reinforcements. Relevant emergent codes (i.e. COVID, Telemedicine, Barriers, Facilitators, etc.) are also included.

EVAR: endovascular aortic repair; HCP: healthcare provider (MD/DO, advanced practice provider); CT: computed tomography scan; US: ultrasound

© 2025 Jarosinski MC et al. *JAMA Network Open.*

eTable 2: Response to Patient Recruitment and Reasons for Declining Study Participation

Response to Recruitment	Patient Follow-up Group	
	**Incomplete**	**Complete**
Completed Interview	12	13
Agreed to/Interested in Interview, Ultimately Not Completed	3	4
Declined Interview (*Patient, nonspecific)*	6	1
Too Many Health Conditions/Obligations	1	2
“Nothing to Contribute”	2	1
Want Approval of Family Physician	0	1
Hard of Hearing/Issues with Technology	1	0
Unable to reach/Incorrect Contact Info	9	12
Deceased (*confirmed)*	7	1
Caregiver Declined Interview on Behalf of Patient (*nonspecific)*	1	0
Too Many Health Conditions, Patient	3	0
**Total:**	45	35

© 2025 Jarosinski MC et al. *JAMA Network Open.*
