# Supplementary material for: Barriers and Facilitators to Endovascular Aortic Repair Follow-Up
Source: JAMA Netw Open. 2025 Dec 2;8(12):e2546327. doi: 10.1001/jamanetworkopen.2025.46327 (PMC12673408; doi:10.1001/jamanetworkopen.2025.46327)
Supplement: Supplement 2. — Data Sharing Statement [file jamanetwopen-e2546327-s002.pdf]

Data Sharing Statement

Jarosinski. Examining Barriers and Facilitators to Endovascular Aortic Repair Follow-Up. *JAMA Netw Open*. Published December 02, 2025. doi:10.1001/jamanetworkopen.2025.46327

Data
Data available: No

Additional Information
Explanation for why data not available: Ensure privacy of a vulnerable patient population
